# They do not have symptoms – why do they need to take medicines? Challenges in tuberculosis preventive treatment among children in Cambodia: a qualitative study

**DOI:** 10.1186/s12890-023-02379-7

**Published:** 2023-03-10

**Authors:** Yom An, Alvin Kuo Jing Teo, Chan Yuda Huot, Sivanna Tieng, Kim Eam Khun, Sok Heng Pheng, Chhenglay Leng, Serongkea Deng, Ngak Song, Daisuke Nonaka, Siyan Yi

**Affiliations:** 1 Sustaining Technical and Analytical Resources (STAR), the Public Health Institute (PHI), Phnom Penh, Cambodia; 2 School of Health Sciences, Faculty of Medicine, University of the Ryukyus, Okinawa, Japan; 3 School of Public Health, National Institute of Public Health, Phnom Penh, Cambodia; 4 Saw Swee Hock School of Public Health, National University of Singapore and National University Health System, Singapore, Singapore; 5 Faculty of Medicine and Health, University of Sydney, Sydney, NSW Australia; 6 National Center for Tuberculosis and Leprosy Control, Phnom Penh, Cambodia; 7 World Health Organization, Phnom Penh, Cambodia; 8 United States Agency for International Development, Phnom Penh, Cambodia; 9 KHANA Center for Population Health Research, Phnom Penh, Cambodia; 10 Center for Global Health Research, Touro University California, Vallejo, CA USA

**Keywords:** Preventive treatment, Childhood tuberculosis, Healthcare providers, Caregivers, Cambodia

## Abstract

**Background:**

Latent tuberculosis (TB) infection has been known as a seedbed for TB disease later in life. The interruption from latent TB infection to TB disease can be done through TB preventive treatment (TPT). In Cambodia, only 40.0% of children under five years old who were the household contacts to bacteriologically confirmed TB cases were initiated with TPT in 2021. Scientific studies of context-specific operational challenges in TPT provision and uptake among children are scarce, particularly in high TB-burden countries. This study identified challenges in TPT provision and uptake among children in Cambodia from the perspective of healthcare providers and caregivers.

**Methods:**

Between October and December 2020, we conducted in-depth interviews with four operational district TB supervisors, four clinicians and four nurses in charge of TB in referral hospitals, four nurses in charge of TB in health centers, and 28 caregivers with children currently or previously on TB treatment or TPT, and those who refused TPT for their eligible children. Data were audio recorded along with field notetaking. After verbatim transcription, data analyses were performed using a thematic approach.

**Results:**

The mean age of healthcare providers and caregivers were 40.19 years (SD 12.0) and 47.9 years (SD 14.6), respectively. Most healthcare providers (93.8%) were male, and 75.0% of caregivers were female. More than one-fourth of caregivers were grandparents, and 25.0% had no formal education. Identified key barriers to TPT implementation among children included TPT side effects, poor adherence to TPT, poor understanding of TPT among caregivers, TPT risk perception among caregivers, TPT’s child-unfriendly formula, TPT supply-chain issues, caregivers’ concern about the effectiveness of TPT, being non-parental caregivers, and poor community engagement.

**Conclusion:**

Findings from this study suggest that the national TB program should provide more TPT training to healthcare providers and strengthen supply chain mechanisms to ensure adequate TPT drug supplies. Improving community awareness of TPT among caregivers should also be intensified. These context-specific interventions will play a crucial role in expanding the TPT program to interrupt the development from latent TB infection to active and ultimately lead to ending TB in the country.

**Supplementary Information:**

The online version contains supplementary material available at 10.1186/s12890-023-02379-7.

## Background

The World Health Organization (WHO) estimates that about 25% of the population globally is infected with tuberculosis (TB) or has latent TB infection, of whom 5–10% will develop active TB during their life course [1]. Children and adolescents are high-risk groups for developing active TB [2]. TB preventive treatment (TPT) can decrease the risk of TB disease in children by 59% [3] and is one of the WHO’s strategies to end TB [4]. Between the 2018 and 2020, 8.7 million people of all ages initiated TPT globally, equalling only 29% of the five-year target (2018–2022) of 30 million set at the United Nations General Assembly High-Level Meeting on TB in 2018 [5].

Children under five years old are among the populations most at risk for progressing from TB infection to TB disease [6], and TPT initiation among them is far behind the target. Only 1.2 million (29%) of the four million under-five children who were household contacts of bacteriologically-confirmed TB cases initiated with TPT between 2018 and 2020 [5]. This low achievement indicates substantial efforts needed to meet the TPT targets in young children.

Several barriers may influence TPT initiation in children. TPT risk perception, lack of information, limited access to TPT, and perceived poor service quality at TPT facilities have been identified as the key barriers to childhood TPT initiation among parents and caregivers [7–10]. Furthermore, caregivers’ personal experience in TPT such as poor adherence and acceptability also affected TPT initiation among children under their care in many countries [11]. From the healthcare provider side, limited knowledge of TPT was a challenge in TPT implementation in Peru [7], India [8, 9, 12], and Kenya [13]. Providers’ perception of TPT’s efficacy and long duration, and lack of standard guidelines and support from the program management level were identified as barriers to TPT initiation in children in Kenya [13]. Healthcare providers reported a lack of screening equipment and expertise to interpret diagnostic results, the high workload for healthcare workers, poor TPT monitoring, and fear of increasing Isoniazid resistance as barriers to child TPT implementation in several settings [8, 9, 11, 13]. In Malawi, transportation costs for chest X-ray screening were the primary reason for low TPT among children younger than six years old [14].

Cambodia is a high TB burden country with an estimated incidence rate of 288 per 100,000 population in 2021 [15] and has been listed as one of three countries on WHO’s global TB watchlist [5]. The country has made enormous efforts through different approaches to reduce this high burden and reach the targets to end TB by 2030 [16]. Providing TPT is among the core interventions [16]. The national TB program has initiated TPT using a nine-month regimen of Isoniazid for children under five years since 2008 [17]. In 2018, the WHO updated and consolidated guidelines for latent TB infection with different choices of TPT regiments [6]. In 2020, Cambodia’s national TB program developed a standard operating procedure (SOP) for latent TB infections and TPT [18] with the national efforts to scale up the TPT implementation. With this SOP, the national program has adopted three regiments, including six months of daily Isoniazid (6H), three months of weekly Isoniazid and Rifapentine (3HP), and three months of daily Isoniazid and Rifampicin (3RH). TPT is prescribed and monitored by healthcare providers at referral hospitals and at health centers.

 TPT initiation among under-five children has fluctuated in the past five years and dropped significantly in 2021, mainly due to the impact of COVID-19. Based on data from  the WHO, the proportion of under-five children eligible for TPT initiated with it was 47% in 2017, 48% in 2019, 89% in 2020, and only 40% in 2021 [15]. While suboptimal TPT implementation was reported, more is needed to understand contextual challenges influencing the implementation. Therefore, it is essential to understand the barriers to providing and receiving TPT for eligible children in the country. This study explored the challenges in TPT provision and uptake among children in Cambodia from the perspective of healthcare providers and caregivers. Findings from this study will provide insights into the challenges of TPT implementation from different angles, which will be beneficial for shaping program implementation and policy development.

## Methods

### Study design, sites, and participants

We conducted this qualitative study between November and December 2020. In-depth interviews (IDIs) were performed with 16 healthcare providers, including four TB supervisors at operational districts (ODs), four clinicians in charge of TB at referral hospitals, four nurses in charge of TB at referral hospitals, and four nurses in charge of TB at health centers under the coverage of the selected ODs. ODs are a functional unit within Cambodia’s health system under the provincial health departments. We also conducted IDIs with 28 caregivers aged ≥ 18 years who had children younger than 15 years old currently or previously with TB (*n=9*), accepted TPT for their eligible children (*n=11*), or rejected TPT for their eligible children (*n=8*).

### Sampling and recruitment

The research team purposively recruited healthcare providers with experience and good knowledge of childhood TB and TPT. The knowledge was measured using a questionnaire on TB causes, transmission routes, signs, and symptoms; characteristics of lymph nodes that implied TB; diagnostic criteria for childhood TB; and TPT use, target groups, drugs, contraindication, and side effects. We have reported the detailed knowledge measures elsewhere [19]. Caregivers were recruited using a convenient sampling method with support from local healthcare providers, community-based non-governmental organizations, and village health support groups. They pre-identified caregivers residing in the coverage area of the selected health facilities with the research team’s guidance.

### Data collection

We collected respondents’ sociodemographic characteristics and perceptions​ of challenges in TPT provision and uptake among children through face-to-face IDIs. Trained and experienced interviewers conducted the IDIs using pre-tested semi-structured interview guides in Khmer with audio recordings and field notes. Key questions for healthcare providers included challenges in providing TPT, such as TPT guideline availability, providers’ TPT knowledge, TPT drug availability, and TPT risk perceptions. For caregivers, the question guide focused on whether they had heard about TPT, thought TPT is safe and effective in TB prevention, and would allow their eligible children to take TPT. We interviewed healthcare providers at TB clinics and caregivers at their houses in private places. Each participant received a compensation gift valued at about one US dollar after the interview which took 30-40 min.

### Data management and analyses

Audio records were transcribed into Khmer and then translated into English. Two researchers (YA and KEK)﻿ manually coded the transcription based on the question guides. Emerged themes and sub-themes on TPT provision and uptake challenges were also coded accordingly. If necessary, identified key themes were verified against Khmer transcription and with the audio records. We analyzed the data using thematic analyses, and pre-identified main themes in the question guides were saturated.

## Results

Table 1 shows the demographic characteristics of the participants. The mean age of healthcare providers was 40.2 (SD 12.0) years, and 93.8% were male. Among the caregiver participants, the mean age was 47.9 (SD 14.6) years, and 75.0% were female. One-fourth of the caregiver participants had no formal education, and 60.7% were farmers.

**Table 1:** Demographic characteristics of in-depth interview participants

	**Frequency**	**%**
**Healthcare providers (*n* = 16)**		
Age in years, mean (SD)	40.2 (12.0)	
Sex, male	15	93.8
Working place		
Operational district	4	25.0
Referral hospital	8	50.0
Health center	4	25.0
Role		
TB supervisor	4	25.0
Clinician at TB service at a referral hospital	4	25.0
Nurse at TB service at a referral hospital	4	25.0
Nurse in charge of TB at a health center	4	25.0
**Caregivers (*n* = 28)**		
Age in years, mean (SD)	47.9 (14.6)	
Sex, female	21	75.0
Relationship of caregivers with children		
Parent	20	71.4
Grandparent	8	28.6
Education		
No formal education	7	25.0
Primary school	8	28.6
Secondary school	10	35.7
High school or higher	3	10.7
Main occupation		
Farmer	17	60.7
Seller	5	17.9
Government or private sector staff	2	7.1
Other	4	14.3

*Abbreviations*: *SD* standard deviation, *TB* tuberculosis

### Barriers to TPT implementation

Healthcare providers and caregivers perceived and experienced several challenges in TPT implementation and uptake among eligible children. The following main themes emerged as barriers.

#### TPT side effects

TPT side effects, such as dizziness, vomiting, blurred vision, and tiredness, were reported by many healthcare providers.

*"They (children) were fine before taking the medicines. But when they took them, it was like killing them… Some children felt normal when they took the medicines. But after one week, they said they weren't well with dizziness, vomiting, etc." (A nurse at a health center, IDI-8, male, 26 years).*

*“Most patients complained that they felt unwell after taking TPT medicines, having nausea, dizziness, blurred vision, or tiredness.” (A doctor at a referral hospital, IDI-11, male, 48 years).*

Caregivers also reported that TPT side effects were the reason for them not accepting TPT for their eligible children.

*“Because* *it had* *side effects*. *I* *was* *afraid my kid* *would* *suffer* *from the side effects.”* *(A* *caregiver who refused TPT for their child, IDI-6, female, 30 years).*

*“…. It was hard to make children take it, and I was worried they were too young to overcome the side effects of the medicines…..” (A caregiver who refused TPT for their child, IDI-1, female, 67 years).*

Some healthcare providers believed that TPT’s side effects, such as vomiting, dizziness, and fatigue, were the primary causes of poor adherence among children. To overcome these side effects, healthcare providers supported caregivers and children by providing medical and psychological support to improve TPT adherence.

*“Yes, when they (children) took the medicines, they had problems with side effects, and they complained and refused to continue taking them.” (A nurse at referral hospital, IDI-14, male, 50 years).*

*“Some children received the TPT, dropped out later, and, in many cases, stopped taking the medicines. At first, we explained to them, and they understood and took the medicines. They continued taking the medicines for about one month, then side effects happened, and they stopped taking them.” (An OD TB supervisor, IDI-1, female, 35 years).*

### Poor understanding of TPT among caregivers

Caregivers raised several reasons for the acceptance or refusal of TPT for their eligible children, including that children were not sick, caregivers’ busyness, or no clear explanation from healthcare providers.

*“We had provided TPT to children or TB close contacts for about a year, but it was still difficult to educate people to bring children in the family who were TB close contacts to receive TPT. Some caregivers said their kids were healthy, so why did they have to receive the treatment? It took quite a long time to make them understood.” (An OD TB supervisor, IDI-13, male, 48 years).*

*“Sometimes, they (caregivers) didn’t accept TPT because they didn’t know (about TPT). Some people were hard to explain; they refused (TPT for their kids) even their family and relatives persuaded them to take the medicines for prevention.” (A nurse at referral hospital, IDI-3, female, 31 years).*

A caregiver also raised a concern that TPT cannot prevent TB disease. Their kids may again get TB if they are in close contact with people with TB.

*“It’s also difficult…suppose we give him TPT up to six months… and then children in the village are infected with TB, and he plays with those children, so he will get the infection again.” (A caregiver who refused TPT for her child, IDI-8, female, 27 years).*

Almost all caregivers who received or refused TPT for their children had heard about TPT and acknowledged its importance and effectiveness. However, several caregivers with children previously or currently on TB treatment admitted that they had never heard about TPT.

*"I had never heard about the medicines* *for preventing TB"* *(A* *caregiver of a child receiving TB treatment, IDI-3, female, 65 years).*

*“Never, never had heard about it* *(TPT)”* *(A* *caregiver of a child receiving TB treatment, IDI-1, female, 25 years).*

Some healthcare providers believed some caregivers did not accept TPT for their eligible children because of misconceptions. They were worried about TPT side effects as they thought their children were not sick and too young and likely to be harmed by the medicines. These misconceptions might be due to unclear explanations from healthcare providers.

*“I used to request parents to bring their children living with adults who had bacteriologically confirmed TB to health facilities for TPT, but the parents were reluctant, maybe because I didn’t explain them well. I didn’t know the specific reason behind that, but they refused it, not willing to take the treatment (A nurse at a health center, IDI-12, male, 28 years).*

*“Like I said before, (challenges in providing TPT) include: first, they (children) were not sick, and we gave them medicines but didn’t explain them clearly. Second, they didn’t want to take the medicines because they were afraid of them, or they didn’t want to take them because the kids were young.” (A doctor at a referral hospital, IDI-16, male, 35 years).*

Some caregivers did not accept TPT because of their perception of TPT’s adverse effects, as their kids were not sick or the medicines could be harmful for the children as they were too young, and there was no clear explanation from healthcare providers.

*“….. but the kid was too young to take the medicines, so I could not make him/her take it. …....If the doctors said it was okay for babies to take the medicines, I would let them take it despite their young age.” (A caregiver who refused TPT for their grandchildren, IDI-1, female, 67 years).*

*“I think that if they have TB, taking medicines is correct. But if they don’t have symptoms, and they must take medicines too, it’s dangerous.” (A caregiver who refused TPT for their children, IDI-4, male, 52 years).*

*“I didn’t* *want him to take medicines* *at* *that* *time because he is young”* *(A* *caregiver who refused* *TPT for her* *child, IDI-8, female, 27 years)*

### Child-unfriendly formula

Caregivers reported that children did not like TPT’s taste, which hindered TPT acceptance among children.

*“My kid couldn’t tolerate its bitterness. Because it was bitter …... if it was a little bit sweet, he might be able to take it.” (A caregiver who refused TPT for her child, IDI-6, female, 30 years).*

### TPT supply issues

Healthcare providers reported inadequate drug supplies interrupted TPT provision.

*"Before,* *there were insufficient drugs, ….. but now no, ….. the challenges before included insufficient drugs (for TPT)*.*"* *(An* *OD TB supervisor, IDI-2, male, 39 years).*

### Being non-parental caregivers

Caregivers who were grandparents reported that the sole reason for not accepting TPT was because the children’s parents were living away from them. It was hard for grandparent caregivers to look after the children when they got sick.

*“Their parents lived somewhere else... It’s hard for me as their caregiver… I looked after all their three children. I didn’t have time to get the medicines for all of them.” (A caregiver who refused to receive TPT for her children, IDI-2, female, 62 years).*

### Improving TPT implementation

When asked how to improve TPT acceptance, most healthcare providers suggested increasing health education on TPT in the community, improving healthcare providers’ capacity to provide TPT, increasing the screening of children for TPT eligibility, and ensuring adequate drug supplies.

*“I think the national* *(TB) program should help spread the information about TPT to make people understand …"* *(An* *OD TB supervisor*, *IDI-7,* *male, 31 years)*.

*"…..first, we have to educate people with TB who have kids and advise them to bring their kids to get TPT after confirming that they do not have TB but are at high risk of having TB. We advise the parents to bring their kids to receive TPT. That is the first strategy. And second, we must advise them (caregivers) to follow the healthcare providers' advice in getting and taking the medicines regularly and try not to miss them to prevent TB transmission." (A doctor at a referral hospital, IDI-9, male, 36 years).*

*"We* *need* *to increase our capacity to* *better understand TPT. We need to know more deeply about it*.*"* *(An* *OD* *TB supervisor*, *IDI-1,* *female, 35 years)*.

## Discussion

This study assessed the challenges in child TPT provision and uptake from the perspective of healthcare providers and caregivers. Perceived TPT side effects were commonly reported as barriers to TPT. These findings were similar to other studies. A study in Lesotho indicated that the fear of TPT’s side effects was the main reason for poor TPT adherence [20]. TPT side effects were also reported as a concern among caregivers and providers in India [8, 21, 22] and Indonesia [23]. To tackle these challenges, it is essential to explain to caregivers the importance of TPT in preventing the progression from TB infection to active TB, as TPT effectively reduces TB incidence by 60% to 90% [24]. Furthermore, healthcare providers’ clear explanation of TPT’s side effects and the availability of TPT with fewer side effects is critical. Cruz and Starke found that 3HP or daily rifampin for four months (4R) had minimal side effects and was well tolerated by children [25].

The TPT knowledge gap among caregivers was also a challenge for TPT provision. This could lead to a high proportion of TPT unacceptance for eligible children. In Southeast Asia, the TPT knowledge gap among service recipients was a reason for the slow TPT scale-up [23, 26–28]. In Australia, 31% of caregivers in the study did not believe in the importance of TB chemoprophylaxis [29]. This misconception might be due to the lack of TPT information in the community or unclear explanations of the TPT effectiveness from healthcare providers. The poor caregivers’ knowledge of TPT was identified as a barrier to TPT initiation in India [8, 9], Ethiopia [30], Rwanda [31, 32], and South Africa [33]. This limited knowledge could also lead to low community participation in screening children for latent TB infection and low TPT provision [20]. In our context, TPT was not prioritized by some caregivers due to their poor understanding of the importance of TPT in preventing active TB in children. Therefore, increasing community awareness of TPT benefits should be prioritized to maximize the uptake.

Unclear explanations about TPT from healthcare providers could raise a concern about the TPT's effectiveness in preventing TB disease among caregivers and may lead to low TPT acceptance for their children. Limited TPT knowledge among healthcare providers is a factor hindering TPT initiation in many settings, such as South Africa [34, 35], Dominican Republic [36], and Kenya [13]. These findings suggest that more comprehensive TPT training for healthcare providers in charge of TB along with TPT community awareness campaigns are essential to tackle this barrier.

Unclear explanations from healthcare providers on the importance of TPT may contribute to this concern which constituted a reason for TPT refusal among caregivers. This finding is similar to a study in India, where children being too young and free of TB symptoms were the reasons for TPT unacceptance, as caregivers had an insufficient understanding of the TPT [8]. Similar findings have also been reported in Indonesia [23, 28], Dominican Republic [36], and Southern Ethiopia [37]. Simple and informative messages on TPT's risks and benefits should be widely communicated through leaflets and community education sessions. The information should include the high risk of developing severe forms of TB disease (e.g., TB meningitis), especially among younger children, if they do not receive TPT.

Inadequate and irregular drug supplies were another challenge for TPT implementation, similar to studies in India [8, 22]. In Southeast Asia, inadequate TPT supplies were a reason for slow TPT scaling-up, which must be urgently addressed [26]. To ensure stable drug supplies, a close and systematic TPT monitoring system from national to sub-national levels should be in place. At the central level, representatives from the central drug store of the Ministry of Health should regularly join quarterly or annual meetings with the national TB program to share experiences and address challenges related to TPT drug supplies. In addition, the national TB program should ensure regular supervision and work closely with relevant partners to ensure adequate drug TPT supplies, monitoring, and distribution [38]. At the sub-national level, OD and provincial TB supervisors should discuss jointly addressing challenges.

This study identified poor collaboration from caregivers as a barrier to childhood TPT implementation. The absence of parents from home was a challenge since caregivers who were grandparents or relatives could not decide to accept TPT for the children. Interventions should be contextualized to the specific reasons and local settings, such as targeted health education among non-parent caregivers on TPT in the community to improve TPT uptake and adherence. The collaboration between caregivers and healthcare providers is key to TPT implementation success. In Nigeria, caregivers lacked collaboration to regularly provide TPT to their children because TPT was less prioritized than other tasks [39]. Therefore, improving engagements between caregivers and the community is a crucial strategy to accelerate TPT implementation and sustainably as they are a key player to enable changes in behavior, environments, and practices within their communities [40].

In Cambodia, TPT is provided to eligible people of all age groups. Challenges to TPT implementation identified in this study may be similar in children and adults. In a survey of TB staff in 35 countries, participants reported a lack of funding to buy TPT drugs, TPT stock-out or TPT supply chain issues, perceived TPT’s adverse effects, and a lack of dedicated staff for the TPT implementation [41]. A situational analysis of TPT program management by the WHO revealed several constraints on TPT implementation in Southeast Asia. The unavailability of TPT drugs, mainly Rifapentine, inadequate TPT healthcare provider training, and inadequate TPT demand from the community were among the challenges in TPT implementation [42]. These challenges could help inform interventions and policies to build a strong TPT program for all age groups in Cambodia.

### Limitations of the study

This study has several limitations. First, self-reported measures may lead to social desirability bias. However, this bias could have been minimized as data collection was conducted by trained and experienced data collectors with health backgrounds. Second, the findings may be unique to the Cambodian setting since identified barriers from this study were contextually and culturally specific. Third, three-fourths of the caregiver participants in this study were women who may experience different challenges in caring for their children from men. Barriers to TPT provision faced by men caregivers may be under-reported. Forth, most healthcare providers participating in this study were male; therefore, barriers to TPT implementation identified in this study may not sufficiently reflect the perspectives of female providers.

Fifth, this study included only healthcare providers with high knowledge of childhood TB and TPT, who may face different challenges from providers with less experience and knowledge. Finally, the convenience sampling method used to recruit caregivers may lead to selection bias since it was done by local healthcare providers, which may lead to bias in the results. However, all caregiver participants were well explained the research objectives and the importance of the research findings in improving TPT implementation in the country. 

## Conclusion

Critical barriers to childhood TPT implementation in Cambodia included perceived TPT’s adverse effects, knowledge gaps among caregivers and healthcare providers, child-unfriendly TPT formula, inadequate and irregular TPT drug supplies, misconception about TPT’s effectiveness among caregivers, absence of parents from home, and poor community engagement in TPT implementation. The national TB program should invest in building healthcare providers’ TPT implementation capacity. Awareness-raising campaigns are critical in improving TPT knowledge and acceptance in the community. Since TPT implementation in Cambodia has recently expanded to all age groups [43], improving TPT implementation in children may also improve the national TPT program and lead to achieving the targets set at the United Nation’s high-level meetings in 2018 [44].

## Supplementary Information

**Additional file 1. **Interview guide for In-Depth Interview (IDI).

## Data Availability

Data and materials are available upon request from Dr. Yom An (Email: anyomniph@gmail.com).
